# SCCNV: A Software Tool for Identifying Copy Number Variation From Single-Cell Whole-Genome Sequencing

**DOI:** 10.3389/fgene.2020.505441

**Published:** 2020-11-16

**Authors:** Xiao Dong, Lei Zhang, Xiaoxiao Hao, Tao Wang, Jan Vijg

**Affiliations:** ^1^Department of Genetics, Albert Einstein College of Medicine, Bronx, NY, United States; ^2^Department of Epidemiology and Population Health, Albert Einstein College of Medicine, Bronx, NY, United States; ^3^Center for Single-Cell Omics in Aging and Disease, School of Public Health, Shanghai Jiao Tong University School of Medicine, Shanghai, China

**Keywords:** single-cell whole-genome sequencing, single-cell whole-genome amplification, amplification bias, copy number variation, software development

## Abstract

Identification of *de novo* copy number variations (CNVs) across the genome in single cells requires single-cell whole-genome amplification (WGA) and sequencing. Although many experimental protocols of amplification methods have been developed, all suffer from uneven distribution of read depth across the genome after sequencing of DNA amplicons, which constrains the usage of conventional CNV calling methodologies. Here, we present SCCNV, a software tool for detecting CNVs from whole genome-amplified single cells. SCCNV is a read-depth based approach with adjustment for the WGA bias. We demonstrate its performance by analyzing data obtained with most of the single-cell amplification methods that have been employed for CNV analysis, including DOP-PCR, MDA, MALBAC, and LIANTI. SCCNV is freely available at https://github.com/biosinodx/SCCNV.

## Introduction

Each single cell in a tissue or cell population has its own unique genome due to accumulating *de novo* mutations, such as single-nucleotide variations (SNVs), structural variations (SVs), copy number variations (CNVs) and aneuploidies. The frequency and spectrum of the mutations reflect the loss of genome integrity of a cell population, critically important to cancer and aging (Vijg and Dong, 2020). To detect the mutations unique to a single cell, single-cell whole-genome sequencing (SCWGS) is necessary. SCWGS requires whole-genome amplification (WGA), which is often biased, leading to uneven distribution of DNA content across the genome or differences between alleles. This essentially constrain the usage of variant callers designed for non-amplified bulk DNA. We recently developed a new software tool, SCcaller, that uses heterozygous SNPs to correct for the allelic bias hampering SNV calling (Dong et al., 2017).

CNV calling is typically based on variation of sequencing depth across the genome. However, for a single cell amplicon, variation of sequencing depth increases dramatically due to the locus-specific amplification bias (Navin et al., 2011; Zong et al., 2012; Chen et al., 2017). To solve this issue computationally, we developed SCCNV, a software tool to identify CNVs from SCWGS. SCCNV is also based on a read-depth approach: it controls not only bias during sequencing and alignment, e.g., bias associated with mappability and GC content, but also the locus-specific amplification bias. We demonstrate the performance of SCCNV using SCWGS data of multiple experimental protocols, i.e., DOP-PCR (degenerative-oligonucleotide PCR), MDA (multiple displacement amplification), MALBAC (multiple annealing and looping–based amplification cycles), and LIANTI (linear amplification via transposon insertion) (Navin et al., 2011; Gundry et al., 2012; Zong et al., 2012; Chen et al., 2017; Dong et al., 2017).

## Materials and Methods

### SCCNV

Our software tool for analyzing single-cell copy number variation (SCCNV) was written in Python. Its source code is freely available with a usage description and an example at Github^1^ under the GNU Affero General Public License v3.0. It uses SCWGS data after alignment as input (i.e., a bam file per single cell). Of note, SCCNV cannot take sequencing data of a pool of single cells (a bam file composed of thousands of single cells data), e.g., the 10× Genomics single-cell copy number data, as input.

First, SCCNV divides the genome into bins of equal size (500 kb as default), and counts the numbers of reads per bin of a cell. This step is relatively time-consuming, and we suggest users to use samtools on a high-performance computer cluster in parallel for all samples to be time-efficient (see instructions on Github). The remaining major steps of SCCNV do not require much computational resources – most modern desktop computers should work well.

SCCNV then normalizes mappability, which indicates the efficiency of the alignment to a genomic region. For a bin *b* of a cell, SCCNV adjusts the raw number of reads, denoted by *NR*_*raw*_, by dividing over the mappability *M*,

(1) $$N\,R_{\text{map},b}=N\,R_{\text{raw},b}\,/\,{\text{M}}_{b}$$

where mappability *M* is a value ranging from 0 to 1. SCCNV uses Encode Align100mer mappability score, downloaded from the UCSC genome browser, and calculates the mappability of each bin by using their weighted average.

Then, SCCNV normalizes for GC content. For a cell, SCCNV calculates the percentile of GC content of each bin. For a bin *b* of the cell, its number of aligned reads after normalizing GC content, *NR*_*GC,b*_, is,

(2) $$N\,R_{G\,C,b}=N\,R_{\text{map},b}\times N\,R_{\mathrm{map}\,,\mathrm{genome}}\,/\,N\,R_{\mathrm{map}\,,b,\mathrm{percentile}}$$

where *NR*_*map,genome*_ is the average *NR*_*map*_ per bin of all bins from the cell; *NR*_*map*_,*_*b*,_*_*percentile*_ is the average *NR*_*map*_ per bin of bins in the same GC percentile as bin *b*.

After normalization for mappability and GC content, a pattern of sequencing read depth emerges that is consistent across different cells amplified using the same experimental protocol, i.e., the locus-specific amplification bias. Therefore, the bias is normalized across all cells in a particular batch and experiment. First, to make the *NR*_*GC,b*_ comparable across cells, SCCNV converts it to a raw copy number estimate, denoted by *CN*_*raw*_*_,b_* for bin *b* of cell *c*, as follows,

(3) $$C\,N_{\text{raw},b,c}=N\,R_{\mathrm{GC}\,,b,c}\,/\,N\,R_{\mathrm{GC}\,,\mathrm{genome},c}\times \text{ploidy}$$

where *NR*_*GC*,__*genome*_*_,c_* is the median *NR*_*GC,c*_ per bin in the genome of cell *c*; ploidy is 2 by default. Second, the adjusted copy number is estimated as,

(4) $$C\,N_{\text{adjusted},b,c}=C\,N_{\mathrm{raw}\,,b,c}\,/\,C\,N_{\mathrm{raw}\,,b,\mathrm{–c}}\times \text{ploidy}$$

where CN_*raw,b,–c*_ denotes the average *CN*_*raw*_ for bin *b* across all cells except cell *c*. Of note, with this step SCCNV aims to discover the difference between the cell *c* and the other cells. When analyzing CNVs of multiple tumor cells, it is not appropriate to use all tumor cells as input of SCCNV; instead, one should use one tumor cell with two or more normal diploid cells as the input.

Then SCCNV uses a sliding window approach to further minimize amplification noise. By default, a window includes 11,500-kb bins, i.e., 5.5 Mb of DNA sequence in total, with a 500-kb step size between two neighboring windows,

(5) $$C\,N_{s\,m\,o\,o\,t\,h\,e\,d,b,c}=\frac{1}{11}\,\sum_{i=b-5}^{b+5}C\,N_{\text{adjusted},i,c}$$

SCCNV then models the distribution of *CN*_*smoothed*_*_,b,c_* of all bins in autosomes of a cell *c* as a normal distribution *N*(μ, σ_*c*_^2^). The μ = 2, and σ is estimated as,

(6) $$\sigma_{c}=\left|C\,N_{\text{smoothed},30.9\%,c}-\mu \right|+\left|C\,N_{\text{smoothed},69.1\%,c}-\mu \right|$$

where *CN*_*smoothed*_,*_30_._9__%,c_* and *CN*_*smoothed*,_*_69_._1__%,c_* are the 30.9 and 69.1% percentiles of the *CN*_*smooth*__*ed*_,*_*b,c*_* of all bins in the autosomes, corresponding to the μ – 0.5σ and μ + 0.5σ percentiles, respectively. Here, we did not use the observed s.d. of *CN*_*smoothed*,_*_*b,c*_* of all the bins because the normal distribution was to estimate amplification noise, not real variation in copy number across the genome. When a cell has several large CNVs, the s.d. will be high, even if its amplification noise remains low.

Assuming equally likely priors, for a bin *b* and a given possible copy number *k* ∈ {0, 1, 2, 3, 4}, its posterior probability is,

(7) $$P\,\left(H_{k}|x\right)=f_{k}\,\left(x\right)\,/\,\sum_{i=0}^{4}f_{k}\,\left(x\right)$$

where *x* is the *CN*_*smoothed*,_*_*b,c*_*, and *f_*i*_(x)* is the probability density function of a normal distribution,

(8) $$f_{i}\,\left(x\right)=\frac{1}{\sigma_{c}\,\sqrt{2\,\pi }}\,\exp\left(-\frac{{\left(x-k\right)}^{2}}{2\,\sigma_{c}^{2}}\right)$$

where the variance σ*_*c*_*^2^ is calculated according to Eq. (5). We only used *k* ∈ {0, 1, 2, 3, 4} because the final copy number call was after multiple testing correction, i.e., Eq. (9) below, and we wished to minimize the number of hypotheses tested, i.e., five for a copy number of 0–4. However, this will result in an underestimation if the real copy number exceeds four. To resolve this issue, for bins with copy numbers ≥4 and ≤100, SCCNV reports the closest integer to the *CN*_*smoothed*,_*_*b,c*_*.

SCCNV allows <1 false positive per cell. Therefore, it determines bin b as a copy number variant when,

$$P\,\left(H_{k}|x\right)\geq 1-\frac{1}{\text{GenomeSize}\left(3.2GB\right)/}=0.998$$

(9) WindowSize⁢(5⁢Mb)

### Sensitivity and False Positive Rate

To determine copy number, SCCNV is based on a statistical test described in equations (8) and (9) for a normal distribution and multiple testing correction separately. With a given value of coefficient of variation (CV) of *CN*_*smoothed*,_*_*b,c*_*, sensitivity and FPR can be estimated as follows. Sensitivity equals the difference between two cumulative distribution functions (CDFs) of Eq. (8) at the upper and lower boundaries, which SCCNV provides the correct CNV call after the correction in Eq. (9). The percentage of FP out of all bins is equal to the sum of (a) CDF at the lower boundary of SCCNV providing an incorrect CN gain call; and (b) 1 – CDF at the upper boundary of SCCNV providing an incorrect CN loss call. Then FPR was estimated as the ratio of % of FP to the sum of % of FP and % of TN.

For example, under the assumption that the true copy number is 2, if SCCNV calls CN = 2 when *CN*_*smoothed*,_*_*b,c*_* is between 1.8 and 2.2, sensitivity = CDF(*x* = 2.2, μ = 2) – CDF(*x* = 1.8, μ = 2), in which CDF is the cumulative distribution function of Eq. (8). If SCCNV calls (a) CN = 1 when *CN*_*smoothed*,_*_*b,c*_* is between 0.8 and 1.2, and (b) CN = 3 when *CN*_*smoothed*,_*_*b,c*_* is between 2.8 and 3.2, then%FP = CDF(*x* = 1,2, μ = 2) + 1 – CDF(2.8, μ = 2).

### Testing Datasets and Preprocessing of Data

Four SCWGS datasets were obtained for demonstrating and validating the performance of SCCNV (Zong et al., 2012; Lodato et al., 2015; Chen et al., 2017; Dong et al., 2017). The datasets included 8.2 TB SCWGS of 63 single human fibroblasts, neurons and cells of a tumor cell line amplified using eight different protocols, i.e., DOP-PCR (Sigma), Rubicon, MALBAC, LIANTI, and MDA (including Qiagen, GE, Lodato et al’s MDA and SCMDA). Supplementary Table 1 lists all the single-cell data used in this study.

Sequence alignment was performed using BWA and GATK as follows (Li and Durbin, 2009; McKenna et al., 2010). Raw sequencing data of each sample (single cell and bulk DNA) were obtained from the SRA database and subjected to quality control using FastQC (version 0.11.4;^2^) and trimming using Trim Galore (version 0.4.1;^3^) with default parameters. Then they were aligned to the human reference genome (version hg19) using BWA MEM (version 0.7.12; option: -t number of CPUs -M reference genome fasta file) (Li and Durbin, 2009). PCR duplications were removed using picard tools (version 1.119;^4^). The alignments were subjected to indel realignment and basepair recalibration using GATK (version 3.5; using options, RealignerTargetCreator, IndelRealigner, BaseRecalibrator, and PrintReads) (McKenna et al., 2010). The step above was used for generating an analysis-ready bam file for other types of variants, e.g., single nucleotide variants, small insertions and deletions, and this step is optional for large CNVs or aneuploidies using SCCNV. Reads with mapQ < 30 were discarded. The number of reads per bin of each sample was calculated using samtools (version 1.3; option: bedcov) (Li et al., 2009). SCCNV (version 1.0) was used to estimate CNV of each cell.

## Results

### Major Steps in SCCNV

**FIGURE 1:**
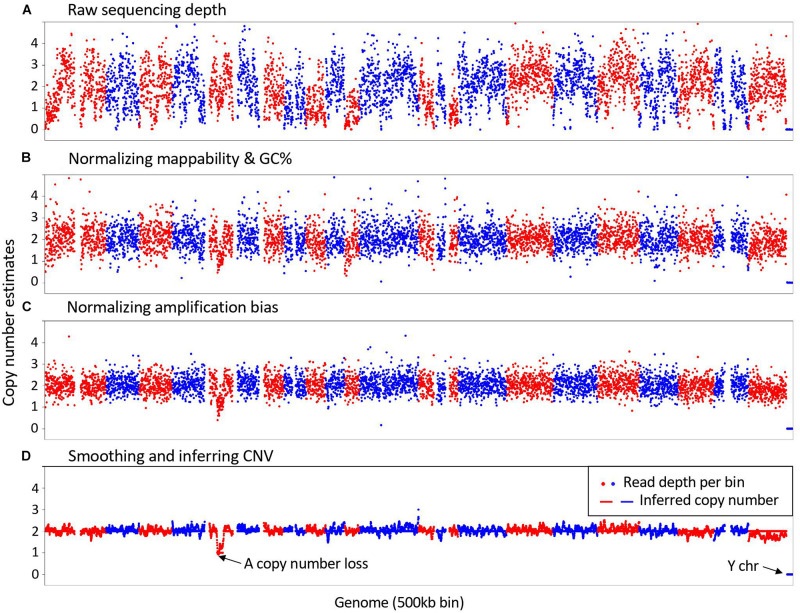
Major steps in SCCNV. A example of copy number estimates of **(A)** Raw sequencing depth; **(B)** after normalizing mappability and GC content; **(C)** after normalizing amplification bias; and **(D)** final results is presented. The example is a normal neuronal nucleus amplified with MDA (SRA id: SRR2141574). Each dot presents a 500 kb bin in the genome. Red and blue colors indicate bins of different chromosomes in Red and blue colors present bins of different chromosomes in their lexicographic order.

As illustrated in Figure 1, SCCNV is composed of four major steps. It first calculates sequencing depth of the genome in bins of equal size (500-kb by default). Second, it normalizes the depth based on two features of the reference genome, including mappability and GC content. These two features are usually also considered by conventional CNV callers for bulk DNA sequencing. Next, it does further normalization across single cells of a same experimental batch. This step minimizes locus-specific bias due to WGA. Finally, it smooths the data (5 Mb by default) and infers copy number of each bin. Intermediate results between any two connecting steps can be generated by SCCNV for users to monitor its performance. We provide an example about the intermediate results of a normal neuronal nucleus amplified with MDA (SRA id: SRR2141574) in Supplementary Figures 1–4.

### Performance on Real Datasets

To evaluate the performance of SCCNV, we obtained four SCWGS datasets from the SRA database, which includes 8.2 TB high-depth WGS data of 63 single human fibroblasts, neurons and cells of a tumor cell line amplified using eight different protocols, i.e., DOP-PCR (Sigma), Rubicon, MALBAC, LIANTI, and MDA (including four MDA protocols, Qiagen, GE, Lodato et al’s MDA and SCMDA) (Supplementary Table 1; Zong et al., 2012; Lodato et al., 2015; Chen et al., 2017; Dong et al., 2017). The data were processed as described in the Materials and Methods.

**FIGURE 2:**
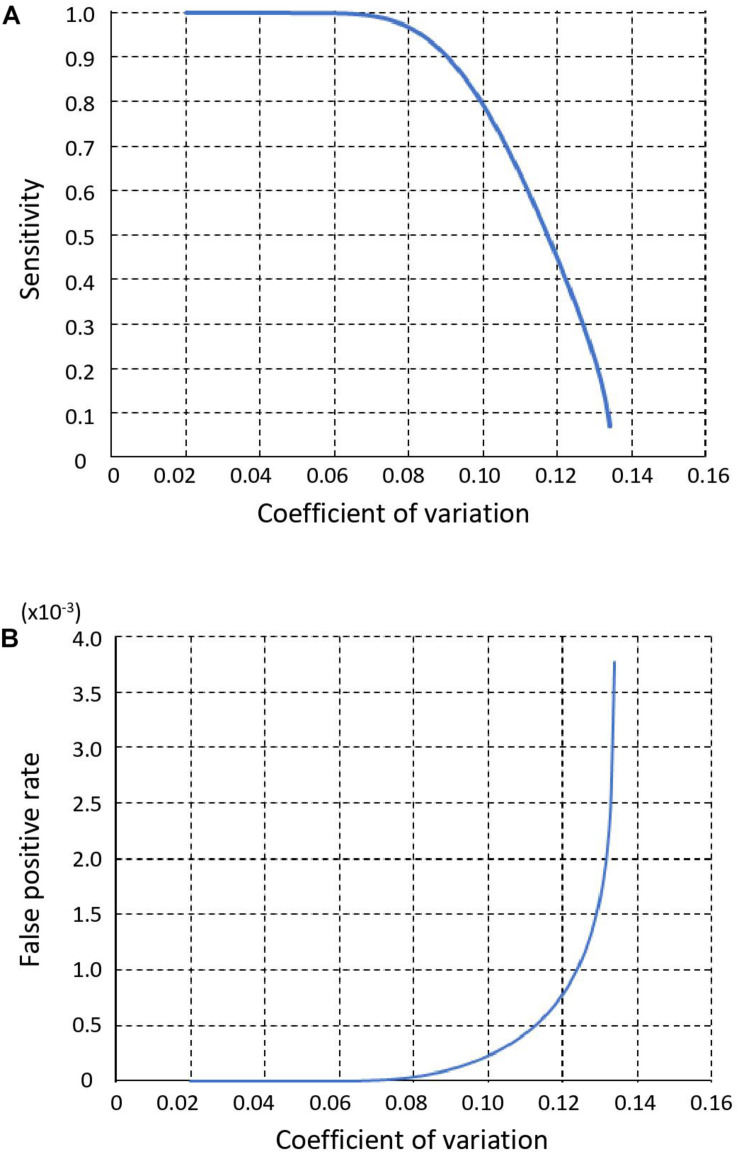
Sensitivity **(A)** and False positive rate **(B)** of SCCNV. *Y*-axis value represents the coefficient of variation of the sequencing read depth (or normalized read depth) of all 500 kb bins across a single-cell genome (see section “Materials and Methods”).

We used the CV of sequencing depth across all genomic bins on autosomes as an indicator of performance, because it directly determines sensitivity and FPR of copy number calling step in SCCNV. We show sensitivity and FPR of the copy number calling in Figures 2A,B, respectively. As the CV decreased from 0.135 to 0.041, the sensitivity increased from 0 to 100% and the FPR decreased from 3.8 × 10^–3^ to 3.9 × 10^–11^.

**FIGURE 3:**
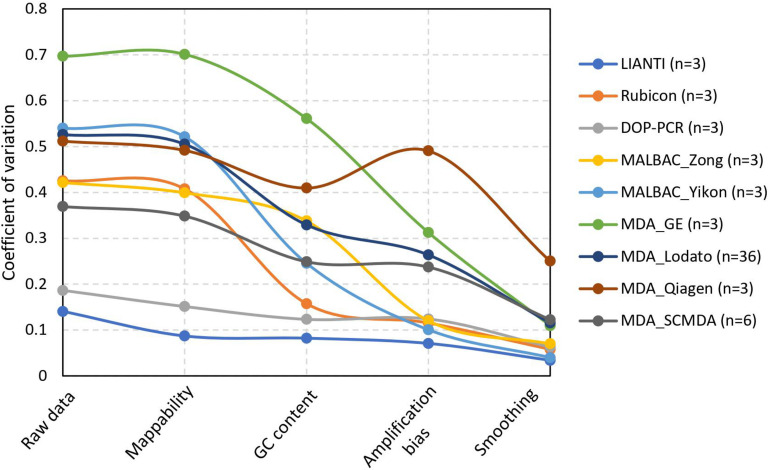
Decreased coefficient of variation by normalization steps in SCCNV. Each line presents the average CV of normalized sequencing depths of multiple single cells amplified using the same experimental procedures. See Supplementary Table 1 for citations and SRA ids of each experimental batch.

For the real datasets, we calculated the CV of raw data and normalized data after each step to demonstrate the performance of normalization in SCCNV (Figure 3). Almost all raw data (CV: 0.475 ± 0.135, avg. ± s.d.) are beyond the detection threshold, i.e., CV = 0.135. Each step of normalization decreased the CV by a significant fraction: on average, mappability normalization by 5%, GC content normalization by 33% percent, across-cell normalization by 22%, and smoothing by 55%. This shows the contributions of each normalization step to performance increase in the final variant calling. After all the normalization steps, the CVs are 0.107 ± 0.076 (avg. ± s.d.), corresponding to a sensitivity of 68.6% and an FPR of 3.6 × 10^–4^ on average. Of note, different amplification protocols have significantly different performance when using SCCNV, likely due to differences in DNA amplification linearity among the protocols. As expected, LIANTI outperformed all the others (Chen et al., 2017). Protocols that included PCR steps, i.e., DOP-PCR, MALBAC and Rubicon, ranked in the middle. Although known as suffering from the least artifactual SNVs (Dong et al., 2017; Zhang et al., 2019), MDA-based protocols were ended last (Figure 3).

## Discussion

Identification of copy number variation and aneuploidy has been one of the major areas of genomics methods development. Several statistical models have been developed for analyzing initially microarray data and later sequencing data of bulk DNA, for example, Circular Binary Segmentation (CBS), Mean Shift-Based (MSB) model, Shifting Level Model (SLM), Expectation Maximization (EM) model, and Hidden Markov Model (HMM) as discussed in Zhao et al. (2013). Based on these models, multiple computational software tools have been developed, e.g., CBS, Copynumber, CNVnator, and HMMcopy (Olshen et al., 2004; Abyzov et al., 2011; Ha et al., 2012; Nilsen et al., 2012). To call CNVs, most of the methods rely on assessing either sequencing read depth or alternate allele fraction at heterozygous SNPs across the genome of one sample, i.e., across-genome normalization. Some of the methods have been applied directly for analyzing single-cell sequencing data with specific filtering for cells with too much bias after WGA.

A few new tools for single cell data were also developed recently under the same rationale (assessing one sample at a time, or across-genome normalization), such as AneuFinder, baseqCNV, Ginkgo and SCOPE (Garvin et al., 2015; Bakker et al., 2016; Fu et al., 2019; Wang et al., 2019). SCCNV was developed based on our observation that the locus-specific amplification bias is often the same in different cells within one experimental batch and amplified using the same protocol (e.g., Supplementary Figure 3); and we showed that normalization across multiple samples (cells) significantly contributed to the increase in variant calling performance for single cells amplified using most WGA protocols (Figure 3). Following the same across-sample normalization rationale, another software tool, SCNV, was developed (Wang et al., 2018). It differs from SCCNV that SCCNV performs normalization based on empirical data directly (Eq. 4) without any assumption on its distribution. Of note, with across-sample normalization, SCCNV essentially aims to identify differences among different cells in one input batch and, therefore, it is important to input cells of interest (e.g., tumor cells) together with cells with a standard diploid genome.

## Conclusion

We developed SCCNV to identify copy number variations from whole-genome amplified single cells. We demonstrated its step-wise performance using most of the recent SCWGS datasets generated with 8 different amplification protocols.

## Data Availability Statement

Raw sequencing data of each sample were obtained from SRA database (SRA SRP067062, SRA SRA060929, SRA SRP102259, SRA SRP041470, and SRA SRP061939).

## Author Contributions

XD, LZ, and JV conceived the study. XD and TW developed the method. XD and XH analyzed the data. XD, LZ, and JV wrote the manuscript. All authors contributed to the article and approved the submitted version.

## Conflict of Interest

XD, LZ, and JV are co-founders of SingulOmics Corp. The remaining authors declare that the research was conducted in the absence of any commercial or financial relationships that could be construed as a potential conflict of interest.

## Footnotes

1 https://github.com/biosinodx/SCCNV

2 https://www.bioinformatics.babraham.ac.uk/projects/fastqc/

3 https://www.bioinformatics.babraham.ac.uk/projects/trim_galore/

4 http://broadinstitute.github.io/picard/index.html

## Code Availability Statement

The source code of SCCNV can be found in the https://github.com/biosinodx/SCCNV.
