# Supplementary material for: SCCNV: A Software Tool for Identifying Copy Number Variation From Single-Cell Whole-Genome Sequencing
Source: Front Genet. 2020 Nov 16;11:505441. doi: 10.3389/fgene.2020.505441 (PMC7701142; doi:10.3389/fgene.2020.505441)
Supplement: Supplementary file 2 [file Data_Sheet_1.PDF]

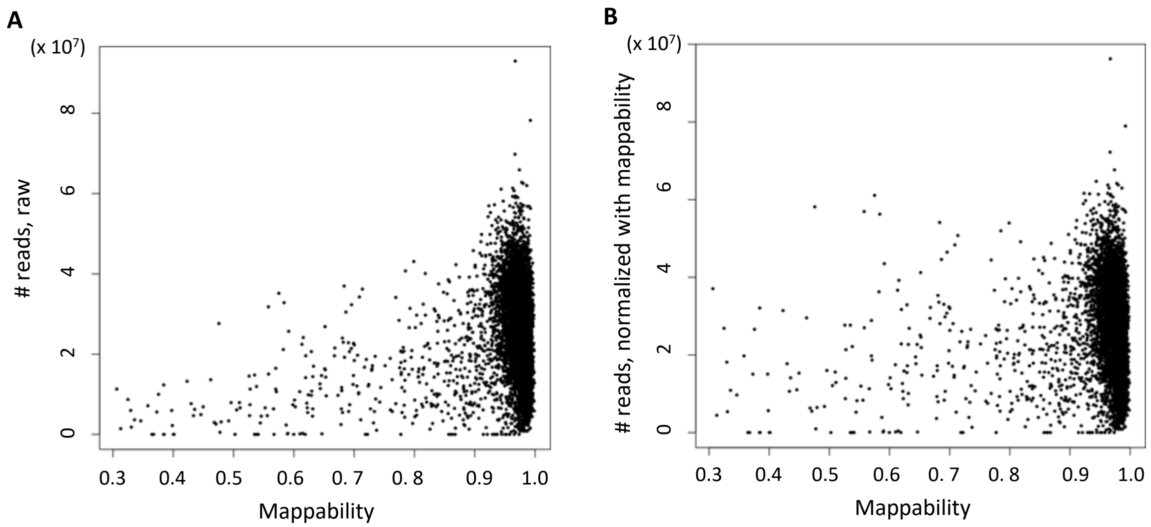

Figure S1. Mappability and number of reads per 500kb bin before (A) and after normalizing (B) the mappability.

The example is a normal neuronal nucleus amplified with MDA (SRA id: SRR2141574). Each dot presents a 500kb bin in the genome. (A) Before the normalization, bins with higher mappability have on average higher read depth. (B) The above bias was corrected after normalization.

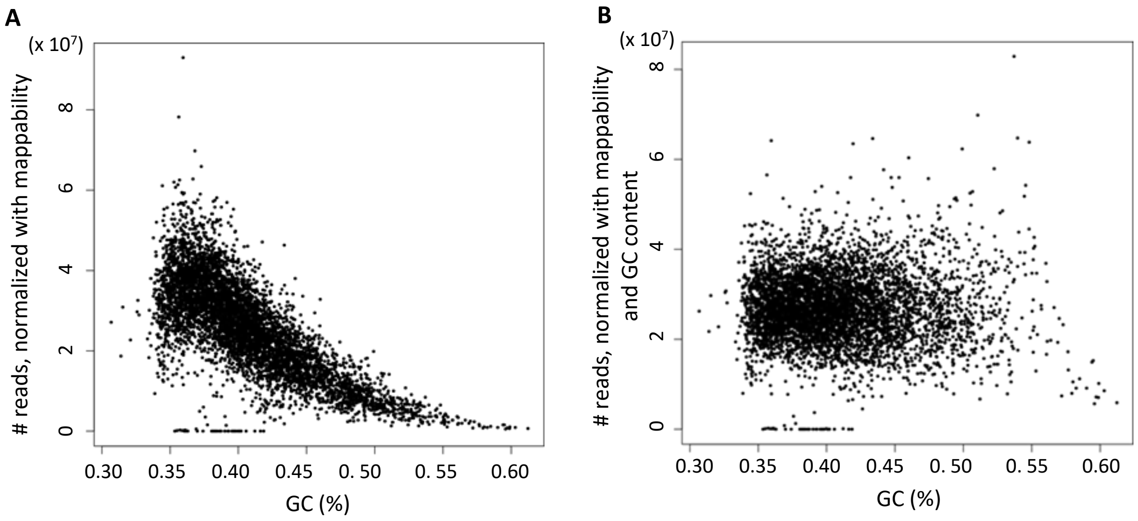

Figure S2. GC content and number of reads per 500kb bin before (A) and after (B) controlling for GC content.

The example is a normal neuronal nucleus amplified with MDA (SRA id: SRR2141574). Each dot presents a 500kb bin in the genome. (A) Before the normalization, bins with different GC % have on average different read depth. (B) The above bias was corrected after normalization.

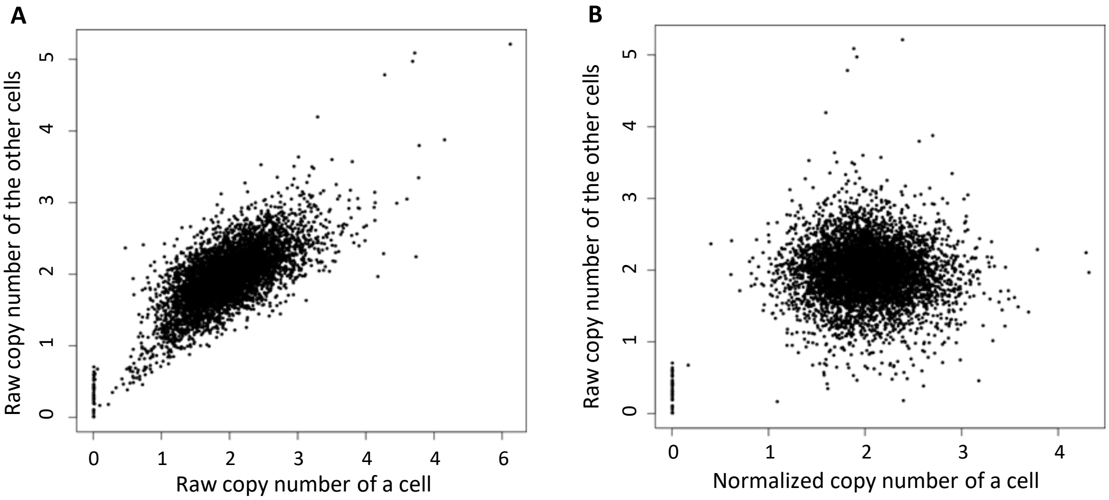

Figure S3. Correlation in copy number estimation between a cell and the other cells of the same batch before (A) and after (B) normalizing locus-specific amplification bias.

The example is a normal neuronal nucleus amplified with MDA (SRA id: SRR2141574). Each dot presents a 500kb bin in the genome. The correlation of bins between uncorrected raw copy number estimation between single cells across the entire genome (as shown in A) was due to consistent locus amplification bias in different single cells.

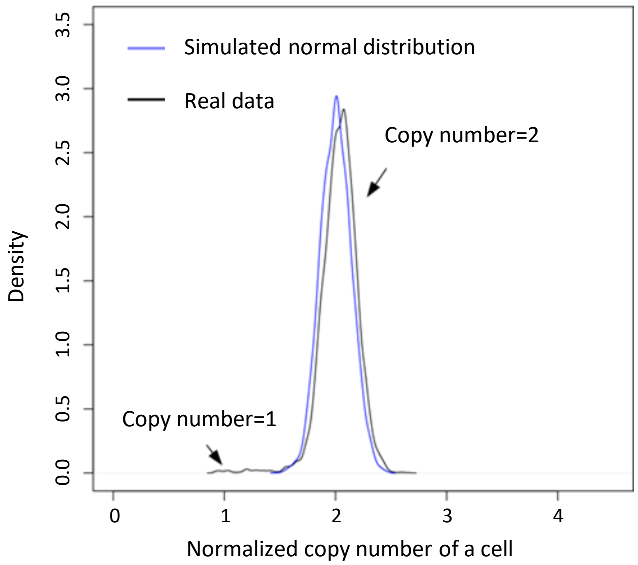

Figure S4. Inferring copy number variation by comparing the observed with a simulated distribution.
The example is a normal neuronal nucleus amplified with MDA (SRA id: SRR2141574).
